# Electron beams probe quantum coherence

**DOI:** 10.1038/s41377-024-01430-4

**Published:** 2024-04-03

**Authors:** Nahid Talebi

**Affiliations:** Institute for Experimental and Applied Physics, Kiel University, 24118 Kiel, Germany

**Keywords:** Optical physics, Optical techniques

## Abstract

Scientists have traditionally employed superimposed mutually-coherent electron beams for holography and phase retrieval of electron wavepackets. However, recent theoretical exploration delves into the interaction of superposed electron beams with the matter. This investigation aims to elucidate long-range Coulomb correlations and quantum decoherence phenomena when electrons interact with their environment.

Quantum coherence stands as a fundamental aspect of quantum technology^1^. Yet, numerous quantum systems are susceptible to decoherence. A thorough examination of decoherence and its influencing factors could accelerate the practical implementation of quantum technological advancements. Theoretically, quantum coherence is depicted by the off-diagonal elements of a quantum system’s density matrix. Experimentally, the visibility of interference fringes resulting from the superposition of two quantum states serves as a gauge for the mutual coherence between them^2^.

Superimposed quantum states may involve either photons or matter waves. Photons typically exhibit minimal interaction with each other, with photon–photon interactions often mediated by the presence of matter, such as atoms, which absorb and emit photons sequentially. Conversely, charged particles like electron beams display strong interactions among themselves and with the environment, facilitated by long-range Coulomb interactions or short-range exchange correlations^3^. This distinctive characteristic renders charged matter waves superior probes of the environment, offering deeper insights into quantum-mechanical decoherence phenomena.

In electron holography experiments, an electron beam is divided into two paths via a biprism^4^. One of the paths passes through a matter, e.g., a thin material film, and both paths are superimposed on a detector screen and form an interference pattern. These interference fringes are analyzed to extract the correlations between the two paths, that can further be used to retrieve the image intensity and phase.

A ground-breaking recent study by Velasco et al.^5^ theoretically examined a special case of long-range Coulomb interactions affecting the superposed electron beams. Electrons interacting with extended objects undergo an inelastic scattering due to the radiative coupling, leading to the exchange of photons with the objects (Fig. 1). This effect has been before experimentally used to probe the decoherence effects in the superposition formed by electron beams^6,7^. This photon exchange can cause a significant loss of the mutual coherence between the two parts of the electron-beam and affect the visibility of the interference fringes observed in the detector^8^. Theoretically, the mutual coherence between the two paths of the electron-beam superposition is modeled by the off-diagonal elements of the distance-dependent density matrix, where the latter is modeled as well by the generalized two-space electron energy-loss probability function integrated over the entire range of photon energies^5^. Intriguingly, although the electron energy-loss probability diverges at ultimately low photon energies, the integration over the entire photon energies remains a constant value. Moreover, as Velasco et al. have demonstrated, electron energy-loss probability function diverges even faster at zero Kelvin temperature compared to a non-zero temperature. Moreover, the rate of photon exchange between the electron beam and the object increases by decreasing the distance *d*_1_ between the beam and the object, leading to a faster yield of the decoherence phenomenon.

**Fig. 1:**
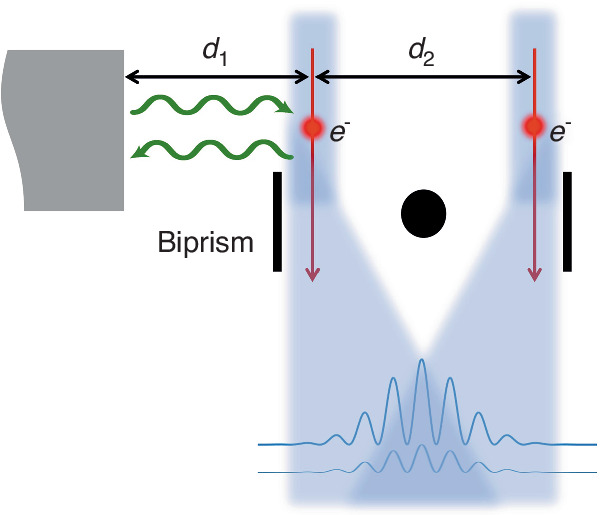
Inelastic interaction due to the radiative coupling between an electron-beam superposition and an extended object, that leads to the decoherence of the mutual correlations between the two electron paths. The decoherence effect leads to the degradation of the visibility of the interference fringes

This work introduced a careful analysis of the decoherence phenomenon for electron-wave interferometry techniques. Next to the recently developed sequential cathodoluminescence spectroscopy that allows for mapping the radiative decoherence channels^9^, the proposed matter-wave scheme allows for the exploration of nonradiative channels and their effects on beam decoherence. Further efforts to extend this formalism to explore the role of different optical medium on the decoherence experienced by electron beams could allow for the realization of schemes leading to the control of the decoherence yield and better understanding of the role that the environment plays on the decoherence phenomenon.
